# Identification of chemosensory genes in the stingless bee *Tetragonisca fiebrigi*

**DOI:** 10.1093/g3journal/jkae060

**Published:** 2024-03-18

**Authors:** María Sol Balbuena, Jose M Latorre-Estivalis, Walter M Farina

**Affiliations:** Laboratorio de Insectos Sociales, Instituto de Fisiología, Biología Molecular y Neurociencias (IFIBYNE), Universidad de Buenos Aires—CONICET, CABA C1428EGA, Argentina; Laboratorio de Insectos Sociales, Departamento de Biodiversidad y Biología Experimental, Facultad de Ciencias Exactas y Naturales, Universidad de Buenos Aires, CABA C1428EGA, Argentina; Laboratorio de Insectos Sociales, Instituto de Fisiología, Biología Molecular y Neurociencias (IFIBYNE), Universidad de Buenos Aires—CONICET, CABA C1428EGA, Argentina; Laboratorio de Insectos Sociales, Departamento de Biodiversidad y Biología Experimental, Facultad de Ciencias Exactas y Naturales, Universidad de Buenos Aires, CABA C1428EGA, Argentina; Laboratorio de Insectos Sociales, Instituto de Fisiología, Biología Molecular y Neurociencias (IFIBYNE), Universidad de Buenos Aires—CONICET, CABA C1428EGA, Argentina; Laboratorio de Insectos Sociales, Departamento de Biodiversidad y Biología Experimental, Facultad de Ciencias Exactas y Naturales, Universidad de Buenos Aires, CABA C1428EGA, Argentina

**Keywords:** stingless bees, *Tetragonisca fiebrigi*, chemosensory genes, transcriptome

## Abstract

Reception of chemical information from the environment is crucial for insects' survival and reproduction. The chemosensory reception mainly occurs by the antennae and mouth parts of the insect, when the stimulus contacts the chemoreceptors located within the sensilla. Chemosensory receptor genes have been well-studied in some social hymenopterans such as ants, honeybees, and wasps. However, although stingless bees are the most representative group of eusocial bees, little is known about their odorant, gustatory, and ionotropic receptor genes. Here, we analyze the transcriptome of the proboscis and antennae of the stingless bee *Tetragonisca fiebrigi*. We identified and annotated 9 gustatory and 15 ionotropic receptors. Regarding the odorant receptors, we identified 204, and we were able to annotate 161 of them. In addition, we compared the chemosensory receptor genes of *T. fiebrigi* with those annotated for other species of Hymenoptera. We found that *T. fiebrigi* showed the largest number of odorant receptors compared with other bees. Genetic expansions were identified in the subfamilies 9-exon, which was also expanded in ants and paper wasps; in G02A, including receptors potentially mediating social behavior; and in GUnC, which has been related to pollen and nectar scent detection. Our study provides the first report of chemosensory receptor genes in *T. fiebrigi* and represents a resource for future molecular and physiological research in this and other stingless bee species.

## Introduction

Insects rely on chemical information (e.g. olfactory, gustatory) from the environment to survive and reproduce. The chemosensory reception of that stimuli occurs mainly by the antennae, mouth parts, and legs when the chemical contacts the chemoreceptors located within specialized structures called sensilla (Scott *et al*. 2001; Robertson and Wanner 2006; de Brito Sanchez 2011; Leal 2013).

Insect odorant receptors (ORs) were first identified in the fruit fly *Drosophila melanogaster*. They are characterized by 7 transmembrane domains and a reverse membrane topology compared with mammalian ORs (Clyne *et al*. 1999). Odorant receptors are expressed in olfactory sensory neurons (OSNs), mostly within trichoid and basiconic sensilla (Leal 2013). Each OR is produced in a subpopulation of OSNs, but all ORs are coexpressed with a chaperoning coreceptor protein called *Orco*, which is involved in localizing ORs to the ciliated dendrites of OSNs and in the signal transduction process (Larsson *et al*. 2004; Vosshall and Hansson 2011). Odorant receptors represent a large and diverse gene family, with no apparent subfamilies or close orthologies across insects, except for *Orco* (Robertson 2019).

In the case of the gustatory receptors (GRs), they are also a family of 7 transmembrane domain proteins that are related to ORs. Gustatory receptors are found in the chemosensory neurons of insects and play a critical role in detecting a wide range of chemicals, including food, mates, and predators (Montell 2009). Likewise, ionotropic receptors (IRs) are a divergent subfamily of ionotropic glutamate receptors (iGluRs) with a similar molecular structure. However, at least 1 of the 3 characteristic residues that interact with glutamate in the iGluR ligand-binding domain is altered in these receptors. Ionotropic receptors are proposed to act as dimers or trimers of subunits coexpressed in the same neuron. These complexes are composed of an odor-specific receptor and 1 or 2 IR coreceptors: *Ir25a*, *Ir8a*, and *Ir76b*. The IRs are distinguished into 2 subfamilies: the conserved “antennal IRs” and the more species-specific “divergent IRs.” The antennal IR subfamily is derived from animal iGluR ancestors and is probably the first olfactory receptor family of insects. The divergent IR subfamily is involved in taste and food assessment and evolved from antennal IRs (Benton *et al*. 2009; Croset *et al*. 2010).

Most of the research about insects' chemosensory receptors (ORs, GRs, and IRs) has been done in the fruit fly *D. melanogaster*. However, several studies have described these receptors in social species of Hymenoptera, mostly in ants (Bonasio *et al*. 2010; Smith, Smith *et al*. 2011; Smith, Zimin *et al*. 2011; Zhou *et al*. 2012; Engsontia *et al*. 2015; McKenzie *et al*. 2016; Slone *et al*. 2017; among others). Regarding eusocial bees, the chemosensory receptors have been well described for the honeybee *Apis mellifera* (Robertson and Wanner 2006; Claudianos *et al*. 2014; Jung *et al*. 2015; Değirmenci *et al*. 2018, 2023; Gómez Ramirez *et al*. 2023), while little is known about stingless bees (Kapheim *et al*. 2015; Brand and Ramírez 2017; Carvalho *et al*. 2017), the most representative group of eusocial bees.

Stingless bees (Meliponini) are important pollinators of tropical and subtropical environments and have economic importance since they are managed for their honey and agricultural purposes (Slaa *et al*. 2006; Grüter 2020). Within corbiculate bees, stingless bees represent the most diverse lineage, with around 600 species (Engel *et al*. 2023); however, a few species have been studied (Grüter 2020). *Tetragonisca fiebrigi* (Schwarz) is a small eusocial stingless bee (4–5 mm) that is found in southwestern Brazil, Paraguay, and Argentina (Castanheira and Contel 2005; Zamudio and Alvarez 2016). Like other insects, eusocial bees rely on olfactory and gustatory information (e.g. floral volatiles, sugar nectar content) to locate and select food sources, to detect and discriminate between enemies and nestmates, and to maintain the organization of the colony (e.g. division of labor). Here, we analyze the transcriptome of the proboscis and antennae of *T. fiebrigi*. We identified the OR, GR, and IR genes, and compared them with the chemosensory receptors annotated for other species of Hymenoptera.

## Materials and methods

### Insects and study site

One colony of *T. fiebrigi* with a queen, brood, and food reserves was used. The hive contained about 3,000 individuals and was open to the field, so bees could forage freely outside. Sampling was done on February 2023 at the Experimental Field of the Universidad de Buenos Aires (Argentina, 34°32′ S, 58°26′ W).

### Tissue dissection and total RNA extraction

Ten foragers (5 non-pollen and 5 pollen foragers) and 10 guards (5 hovering guards and 5 standing guards) were collected from the nest entrance. We identified each group of workers according to their behavior. Foragers were captured immediately before entering the nest. Pollen foragers were identified by the pollen loads on their hind legs and non-pollen foragers by their distended abdomen and the lack of pollen on their hind legs. Guard bees were collected directly from the nest entrance tube, standing guards, or while hovering in front of the entrance tube, hovering guards.

Bees were anesthetized by placing them on ice until they were immobile, and then their antennae and proboscis were dissected using a stereomicroscope and immediately included in 100 μL of Trizol (Invitrogen, Carlsbad, CA, USA). Total RNA was extracted using a pool of 40 antennae (10 antennae per group) and 20 proboscis (5 proboscis per group) following the manufacturer’s specifications. The integrity of the extracted RNA was assessed using 1% agarose gel electrophoresis and quantified using a NanoDrop 1000 spectrophotometer (Thermo-Fisher Scientific, Waltham, MA, USA).

### Sequencing and read processing

Library construction and sequencing services were carried out by Novogene Corporation, Inc. (Sacramento, CA, USA). One cDNA library, using RNA extracted from the pool of antennae and proboscis, was generated using the TruSeq Stranded mRNA LT Sample Prep Kit from Illumina (San Diego, CA, USA) and sequenced using Illumina NovaSeq 6000 equipment with paired-end reads of 150 base-pair (bp) length. The raw data set is available at Sequence Read Archive (SRA) with BioProject number PRJNA1021589.

FastQC v0.11.5 software tool (http://www.bioinformatics.babraham.ac.uk/projects/fastqc/) was used to analyze raw read quality and detect the presence of Illumina adaptors. Following, Trimmomatic v0.36 was used to trim off low-quality bases using the parameters: *trailing*, 5; *leading*, 5; and *sliding-window*, 4:15. Those reads shorter than 50 bp and adaptors were removed using the *ILLUMINACLIP: TruSeq3-PE-2.fa:2:30:10* parameter.

### Transcriptome assembly and prediction of coding sequences

The trimmed and cleaned reads generated by Trimmomatic were used to generate a de novo assembly in the Galaxy Platform by Trinity v2.8.5 (The Galaxy Community 2022), with the option *–SS_lib_type RF* used for stranded libraries and a minimum contig length of 100 bp. The *TrinityStats.pl* script was then used to obtain the basic statistics of the assembly. A non-redundant coding sequence (CDS) database of the assembled transcripts was accomplished following the strategy used by Traverso *et al*. (2022) and Latorre-Estivalis *et al*. (2022). First, open reading frames (ORFs) of at least 100 amino acid lengths were predicted using the *TransDecoder.LongOrfs* script from the TransDecoder v5.5.0 (http://transdecoder.github.io). Second, BLASTp v2.9.0+ and HMMscan v3.2 searches were conducted on the predicted ORFs using the complete UniProtKB/Swiss-Prot and Pfam-A (Bateman et al. 2023; Bairoch and Amos 2000; Mistry et al. 2021) databases as queries. The output of these searches was used in the *TransDecoder.Predict* script to produce the predicted CDSs. Next, CDSs with a sequence identity > 90% were grouped into clusters, keeping only 1 representative sequence per cluster using the *cd-hit-est* script of CD-HIT v.4.8.1. The resulting non-redundant CDS database was translated to amino acids and used to identify the OR, IR, and GR genes of *T. fiebrigi.* The completeness of the non-redundant translated CDS database was analyzed through BUSCO v5.4.6, in protein mode, against the hymenoptera_odb10 data set.

### Identification of chemosensory receptors

BLASTp searches on the non-redundant translated CDS database were performed using the following queries: (1) the OR, GR, and IR sequences from *A. mellifera*, *Bombus terrestris*, *Euglossa dilemma*, *Lasioglossum albipes*, and *Melipona quadrifasciata* and (2) the PFAM seed sequences in fasta format (unaligned) for the 3 families. In the case of the OR BLAST searches, sequences from the following species were also included: 2 solitary wasps (*Ceratosolen solmsi* and *Microplitis demolitor*) and 5 ants (*Acromyrmex echinatior*, *Atta cephalotes*, *Cardiocondyla obscurior*, *Monomorium pharaonis*, and *Solenopsis invicta*) from Zhou *et al*. (2015); 5 *Polistes* wasps (*Polistes fuscatus*, *Polistes metricus*, *Polistes dorsalis*, *Polistes canadensis*, and *Polistes dominula*) from Legan *et al*. (2021); and 21 species from different orders analyzed by Mier *et al*. (2022). BLAST results were filtered with a minimum sequence identity of 20%, an e-value < 1 × 10^−7^ and a minimum alignment length of 200 amino acids. Additionally, HMMscan searches using HMM PFAM profiles for each target family were executed on the non-redundant translated CDS database to identify additional candidates. Finally, candidate sequences were analyzed by (1) BLASTp searches against UniProtKB/Swiss-Prot, (2) HMMscan searches using the Pfam-A database as query, and (3) the presence of transmembrane domains using the TMHMM software v2.0. The results were integrated using Trinotate v3.2.1 (https://trinotate.github.io). Those sequences that do not have the representative PFAM domain of each family were eliminated. Using BLASTp searches against the non-redundant (nr) protein sequence database from the National Center for Biotechnology Information (NCBI), *T. fiebrigi* sequences were manually examined to determine if they were fragments of other predictions (in this case, they were eliminated), if they presented assembled errors (e.g. a sequence was split into 2 assembled transcripts), or if their N- and C-terminus were truncated when 20 or more amino acids were missed (they were classified as partial). These manually curated sequences were used for the phylogenetic analyses described below.

The OR, GR, and IR genes of *Megalopta genalis* were identified using Bitacora v1.3 (Vizueta *et al*. 2020) in “full” mode over genome sequences (USU_MGEN_1.2) and their protein annotations available at https://www.ncbi.nlm.nih.gov/datasets/genome/GCF_011865705.1/. In the case of *L. albipes* IRs, they were identified using Bitacora in “genome” mode over the genome sequences (ASM34657v1) available at https://www.ncbi.nlm.nih.gov/datasets/genome/GCA_000346575.1/. In both species, only those sequences longer than 200 amino acids were considered candidates.

### Phylogenetic analyses

The protein sequences were aligned using MAFFT with the G-INS-i strategy. Afterward, the alignments were trimmed using trimAl v1.2 by default, except for the gap threshold = 0.3. These alignments were then used to build the phylogenetic trees with IQ-tree v1.6.12. The branch support was estimated using both the approximate likelihood ratio test based on the Shimodaira–Hasegawa (aLRT-SH) and the ultrafast bootstrap (UFBoot) approximations (Hordijk and Gascuel 2005). ModelFinder was used to establish the best-fit amino acid substitution models and select based on the Bayesian information criterion. These models were JTT+F+R7 for ORs, JTT+F+R5 for GRs, and JTT+F+R8 for IRs. The phylogenetic trees were visualized and edited with iTOL (Letunic and Bork 2021). Gene candidates of *T. fiebrigi* were annotated based on their relationship with those of *M. quadrifasciata*. *Tetragonisca fiebrigi* candidates without a clear relationship retained their Trinity codes from the assembly. The OR subfamilies were annotated following the classification described by Brand and Ramírez (2017). All *T. fiebrigi* protein sequences and those from other insects used in this work are available in fasta format in Supplementary File 1.

## Results

### Sequencing and de novo assembly

A total of 43.94 M raw reads were obtained. After the trimming and cleaning process, we got 42.78 M reads that were used to generate the assembled transcriptome that contained a total of 321,324 transcripts, with a GC percent of 39.46. The N50 value of contigs in the assembled transcriptome was 1,704 bp, while the median contig length reached 241 bp, and the average contig was 583 bp. A total of 63,400 predicted CDSs were identified, and 23,895 of them were maintained after filtering by redundancy. The BUSCO searches (v5.5.0) based on this data set revealed 84.5% of completeness.

### ORs

A total of 204 ORs were identified in the transcriptome of *T. fiebrigi*, with an average length of 358 amino acids (ranging from 203 to 636) (Supplementary Table 1). A total of 122 (60%) had their sequences complete, while 82 (40%) presented partial sequences. Only 10 sequences do not have the PFAM domain PF02949.23 which is characteristic of the OR family. Besides, 120 sequences (59%) were mapped against an insect OR from the UniProtKB/Swiss-Prot database. A total of 164 (80%) ORs had between 4 and 8 transmembrane domains. The OR repertoire of *T. fiebrigi* (204) is the largest of the 7 species of hymenopterans here analyzed, together with the other stingless bee *M. quadrifasciata* (196) (Table 1). In the case of *M. genalis*, 130 ORs were identified (Table 1): 43 news receptors not predicted in the genome annotation (identified as MgenOr-gene(g)+number), and the rest of the OR sequences were obtained from the predicted protein database (identified as Mgen_Protein ID from GenBank).

**Table 1.** Number of OR, GR, and IR receptors reported in different species of Hymenoptera.

Species	Group	ORs	GRs	IRs	Data origin	References
*A. mellifera*	Honeybee—corbiculate bee	177	13	10	Genome	Robertson and Wanner (2006)
*B. terrestris*	Bumble bee—corbiculate bee	166	25	21	Genome	Sadd *et al*. (2015)
*T. fiebrigi*	Stingless bee—corbiculate bee	204*^a^*	9*^a^*	15*^a^*	Transcriptome	Present study
*M. quadrifasciata*	Stingless bee—corbiculate bee	196	16	10	Genome	Kapheim *et al*. (2015), Brand and Ramírez (2017)
*E. dilemma*	Orchid bee—corbiculate bee	183	13	9	Genome	Brand and Ramírez (2017)
*L. albipes*	Halictid bee—noncorbiculate bee	158	23	22*^a^*	Genome	Kocher *et al*. (2013)
*M. genalis*	Halictid bee—noncorbiculate bee	130*^a^*	29*^a^*	28*^a^*	Genome	Kapheim *et al*. (2015)
*H. saltator*	Ant	377	21	23	Genome	Zhou *et al*. (2012)
*C. floridanus*	Ant	407	63	31	Genome	Zhou *et al*. (2012)

^*a*^Genes identified in this study.

**Fig. 1.**
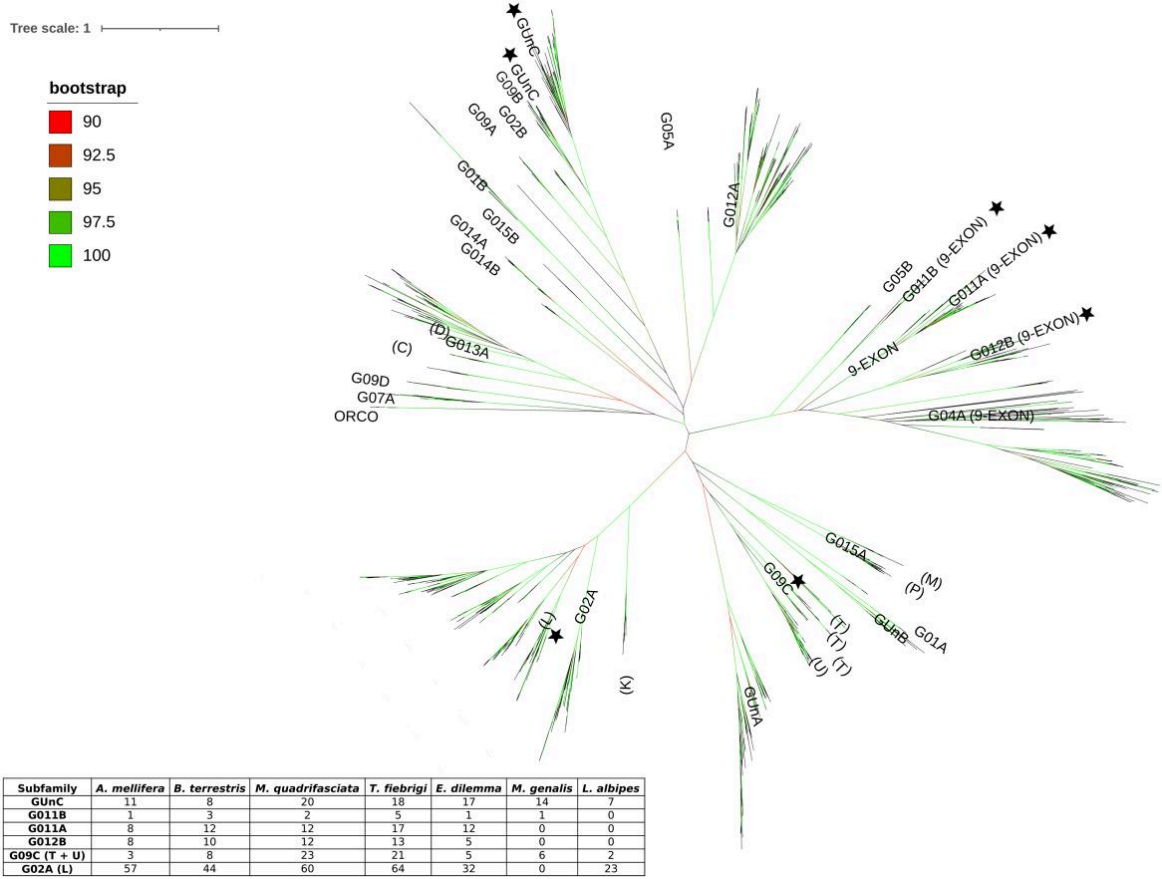
Odorant receptor phylogenetic tree. The maximum likelihood tree was constructed using IQ-tree, and bootstrap support corresponds to aLRT-SH values. Odorant receptor subfamilies were identified following the classification proposed by Brand and Ramírez (2017). Black stars indicate those subfamilies expanded in *T. fiebrigi*. The inserted table shows the number of ORs of those subfamilies across different species of Hymenoptera.

We were able to annotate 161 out of 204 *T. fiebrigi* ORs based on their phylogenetic relations to *M. quadrifasciata* (Supplementary Fig. 1). In most cases, the *T. fiebrigi* ORs were grouped with those of *M. quadrifasciata*. The phylogenetic tree rooted in the conserved *Orco* proteins showed a complex relationship between the specific ORs across the 7 bee species analyzed here. The phylogenetic tree presented 5 large, and well-supported clades containing the 25 OR subfamilies previously described for hymenopterans (Fig. 1). Our analysis was consistent with Brand and Ramírez (2017) and Legan *et al*. (2021), with a few exceptions: G05A subfamily was clustered with G012A, while the G01B and G014B subfamilies were located together with GUnC, G09A, G09B, G014A, G015B, and G02B (Fig. 1 and Supplementary Fig. 1). The ORs were distributed in homologous OR orthogroups consisting of orthologs and/or inparalogs (i.e. lineage-specific expansions). Of these orthogroups, 12 were simple 1:1 orthologous genes that were conserved in 4 or more species studied here, for example, those from G09B, G09D, G01A, or G02B subfamilies, among others (Table 2 and Supplementary Fig. 1). The rest of the orthogroups included species-specific, tribe-specific, or genus-specific expansions. The G02A subfamily (formed by the L and K subfamilies) was the largest (292 ORs across the 7 species), with expanded repertoires in all eusocial bee species (*A. mellifera*, 59 ORs; *B. terrestris*, 46 ORs; *T. fiebrigi*, 66 ORs; and *M. quadrifasciata*, 62 ORs) compared with 33 ORs in *E. dilemma*, 25 ORs in *L. albipes*, and 1 OR detected in *M. genalis* (Table 2 and Supplementary Fig. 1).

**Table 2.** Number of Odorant receptors according to subfamilies, present in different species of Hymenoptera. Odorant receptor subfamilies were identified following Brand and Ramírez (2017) and Zhou *et al*. (2012) classifications. See Supplementary Fig. 1 for more information.

Classification according to		Species						
Brand and Ramírez (2017)	Zhou *et al*. (2012)	*A. mellifera*	*B. terrestris*	*M. quadrifasciata*	*T. fiebrigi^a^*	*E. dilemma*	*M. genalis^a^*	*L. albipes*
G07A	A	3	1	2	4	2	1	1
G09D	B	1	1	1	1	1	1	1
G013A	C	1	1	1	1	0	1	1
	E	6	11	7	7	5	25	24
G01B	F	1	1	0	0	0	6	2
G09A	G	3	1	1	1	3	0	0
G02B	H	1	1	1	1	1	1	1
G09B	H	1	2	1	1	1	0	0
GUnC	H	11	8	20	18	17	14	7
G15B	—	1	1	0	1	0	0	0
G14A	—	1	4	5	3	1	1	1
G14B	W	1	1	1	1	1	1	1
G05A	I	1	1	1	2	1	1	1
G012A	J	21	16	20	18	41	16	14
G05B	—	0	1	1	1	0	1	1
G011B	9-exon	1	3	2	5	1	1	0
G011A	9-exon	8	12	12	17	12	0	0
G012B	9-exon	8	10	12	13	5	0	0
G04A	9-exon	24	11	1	9	8	18	40
**Total 9-exon**		41	36	27	44	26	19	40
G015A	M	1	1	1	1	1	0	1
	P	5	10	1	0	1	0	8
G01A	Q	1	1	1	1	1	1	1
GUnB	—	0	0	1	1	2	14	6
G09C	T	2	3	4	8	3	5	5
	U	1	5	19	13	2	1	2
GUnA	V	6	7	5	9	21	19	13
G02A	L	57	44	60	64	32	0	23
	K	2	2	2	2	1	1	2
ORCO	—	1	1	1	1	1	1	1
**LalbOr10** *^b^*		0	0	0	0	0	0	1
**Sum**		170	161	184	204	165	130	158

D, N, O, R, and S subfamilies are not present in *A. mellifera*.

^*a*^Genes identified in this study.

^*b*^Unclassified sequence.

*Lasioglossum albipes* and *M. genalis* presented expanded lineages in the G013A (25 and 26 ORs, respectively, vs 40 ORs from the sum of the other 5 species), GUnA (13 ORs for *L. albipes* and 19 for *M. genalis*), and GUnB subfamilies (20 ORs from both species and only 4 ORs from the rest of subfamilies (Table 2 and Supplementary Fig. 1). Any OR from *L. albipes* and *M. genalis* was detected in the G011A, G012B, and G015B subfamilies (Table 2 and Supplementary Fig. 1). The stingless bees had expansions in the G09C subfamily, with 23 ORs for *M. quadrifasciata* and 21 for *T. fiebrigi*; in the G011A subfamily, with 12 ORs for *M. quadrifasciata* and 17 ORs for *T. fiebrigi*; and in the GUnC subfamily with 20 ORs for *M. quadrifasciata* and 18 ORs for *T. fiebrigi* (Table 2 and Supplementary Fig. 1). *Megalopta genalis* presented an exclusive expanded clade in the G01B subfamily (6 ORs), and it was absent in the G09A, G09B, G011A, G011B, G015A, and G015B subfamilies. The repertoire of *E. dilemma* was expanded in GUnA (21 ORs), G012A (41 ORs), and G011A (12 ORs) subfamilies (Table 2 and Supplementary Fig. 1). *Apis mellifera* presented the largest species-specific expansions with 17 ORs (*AmelOr122-138*) and 14 ORs (*AmelOr36-49*) in the G04A and G02A subfamilies, respectively (Supplementary Fig. 1). Our results confirmed that the evolution of the OR family within bees is highly dynamic and characterized by lineage-specific gene expansions.

### GRs

**Fig. 2.**
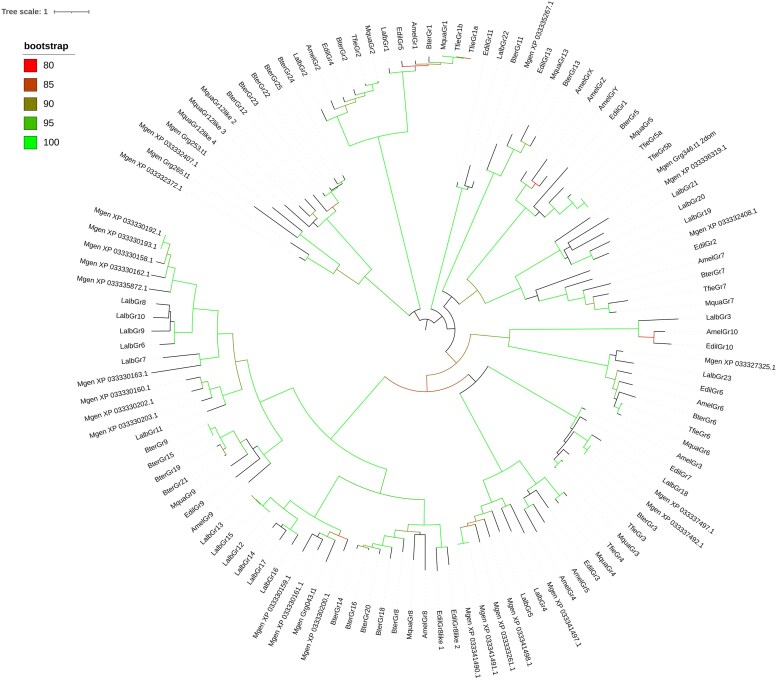
Gustatory receptor phylogenetic tree. The maximum likelihood tree was constructed using IQ-tree, and bootstrap support corresponds to aLRT-SH values. The tree was rooted at the midpoint. Amel, *A. mellifera*; Bter, *B. terrestris*; Edil, *E. dilemma*; Lalb, *L. albipes*; Tfie, *T. fiebrigi*; Mgen, *M. genalis*; and Mqua, *M. quadrifasciata*. Sequences with Trinity IDs belong to *T. fiebrigi.*

The data mining of our transcriptome allowed the identification of 9 GRs, with an average length of 399 amino acids, and only 4 of them were incomplete transcripts (Supplementary Table 1). The number of transmembrane domains of the *T. fiebrigi* GRs varied from 4 to 7. Only 1 GR did not show a positive hit against PF08395.15 and PF06151.16 domains, which are the PFAM domains of the GR family. We were able to assign an annotation to all the *T. fiebrigi* GRs (Fig. 2). In the case of *M. genalis*, 29 GRs were identified, including 4 new receptors that were not predicted in the genome annotation. The GR repertoire of *T. fiebrigi* is the shortest (9) while *M. genalis*, *L. albipes*, and *B. terrestris* presented the highest number of GRs with 29, 23, and 25 receptors, respectively (Table 1).

The phylogenetic tree did not reveal any GR expansion for *T. fiebrigi*, while 2 potential isoforms were identified for *TfieGr1* and *TfieGr5* (Fig. 2). The orthologs of the known sugar receptor genes (*Gr1* and *Gr2*) and another insect-wide conserved (*Gr3*) were found for the 7 species studied here. These lineages, as well as *Gr6*, displayed a simple 1:1 ortholog relationship. The *Gr7* and *Gr4* orthogroups were also conserved, but *L. albipes* and *M. genalis* had expansions between 3 and 5 genes. Finally, none of the *T. fiebrigi* GRs were identified within the *Gr8* and *Gr9* orthogroups, for which *B. terrestris* and the halictid bees, *L. albipes* and *M. genalis,* present species-specific expansions that vary between 4 and 6 paralogs.

### IRs

A total of 15 IRs were identified for *T. fiebrigi* (Supplementary Table 1). Nine of them had complete sequences, while the remaining showed partial sequences. A total of 8 sequences had the PFAM domains PF00060.29 and PF10613.12 typical of this receptor gene family. Ten sequences presented 3 or 4 transmembrane domains. Twenty-two and 28 IRs were identified for *L. albipes* and *M. genalis*, respectively (Table 1).

**Fig. 3.**
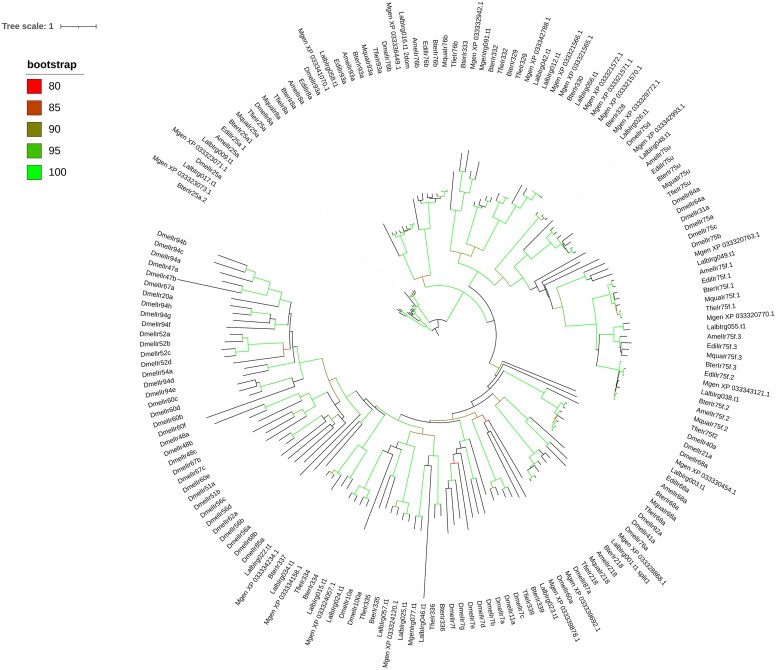
Ionotropic receptor phylogenetic tree. The maximum likelihood tree was constructed using IQ-tree, and bootstrap support corresponds to aLRT-SH values. The tree was rooted using the *Ir25a* and *Ir8a* sequences. *Drosophila melanogaster* sequences were obtained from Croset *et al*. (2010). Amel, *A. mellifera*; Bter, *B. terrestris*; Dmel, *D. melanogaster*; Edil, *E. dilemma*; Lalb, *L. albipes*; Tfie, *T. fiebrigi*; Mgen, *M. genalis*, and Mqua, *M. quadrifasciata*. Sequences with Trinity IDs belong to *T. fiebrigi.*

The three IR coreceptors *Ir25a*, *Ir8a*, and *Ir76b*, as well as the orthologs of the antennal IRs *Ir68a* and *Ir93a* (Fig. 3) were identified. However, other conserved IRs, including *Ir31a*, *Ir92a*, *Ir41a*, *Ir40a*, *Ir76a*, *Ir64a*, *Ir75c*, *Ir75d*, and *Ir84a*, were not detected for any bee species. Most IR clades had 1 sequence per species, except for *Ir330* where *M. genalis* and *L. albipes* present 5 and 2 paralogs, respectively. Within the Ir75 lineage, we identified 4 conserved and separated orthogroups (*Ir75u*, *Ir75f1*, *Ir75f2*, and *Ir75f3*), present in all bee species analyzed. Regarding the other lineages, only the *Ir218* had a member from each of the 7 bee species, while the rest (*Ir328-330*, *Ir332-337*, and *Ir339*) only had sequences from *B. terrestris*, *L. albipes*, *M. genalis*, and *T. fiebrigi.* The differences in these clades were reflected in the reduced IR repertoires observed for *A. mellifera*, *M. quadrifasciata*, and *E. dilemma* (Table 1).

## Discussion

We have annotated and studied 3 sensory receptor gene families (ORs, GRs, and IRs) of the stingless bee *T. fiebrigi* using a de novo assembly generated from antennae and proboscis sequenced transcripts. In parallel, these protein families were identified in the genome of the halictid bee *M. genalis*. Additionally, the sensory repertoires of these 2 species were studied at the evolutionary level along with the repertoires of the eusocial bees *A. mellifera*, *B. terrestris*, and *M. quadrifasciata*, the halictid bee *L. albipes*, and the orchid bee *E. dilemma*.

*Tetragonisca fiebrigi* presented the largest OR (204) repertoire among the bees studied here (Table 2). Compared with other hymenopterans, *T. fiebrigi* and wasps (Legan *et al*. 2021) present a similar number of ORs, which is only surpassed by ants such as *Harpegnathos saltator* (377) and *Camponotus floridanus* (407) (Zhou *et al*. 2012). We manually revised all *T. fiebrigi* OR sequences, verified their proper identification, and provided their annotation. Nevertheless, some of these receptors had partial sequences; hence, the definitive repertoire will be available by analyzing a future *T. fiebrigi* genome.

The high number of ORs found for *T. fiebrigi* is mainly explained by the genetic expansions on the 9-exon, G02A, and G09C subfamilies (Fig. 1, Table 2, and Supplementary Fig. 1). Comparing the 2 species of stingless bees analyzed here, the expansions on G011B (5 genes) and G04A (9 genes) lineages from 9-exon seem to be a unique feature of *T. fiebrigi*, as *M. quadrifasciata* showed fewer receptors in these subfamilies (2 genes in G011B and 1 gene in G04A). The 9-exon subfamily also appears as an expanded clade in several ant species (*Cerapachys biroi*, *C. floridanus*, *A. echinatior*, and *A. cephalotes*; Engsontia *et al*. 2015) and *Polistes* paper wasps (Legan *et al*. 2021). It has been proposed that these receptors detect cuticular hydrocarbons (CHCs) in hymenopterans, compounds that play a key role in nestmate recognition in those species. Interestingly, Balbuena *et al*. (2018) found that the CHC profiles of workers of *T. fiebrigi* differ according to the tasks they perform (e.g. foraging and guarding), which could play an important role in the coordination and cohesion of the colony. Thus, our finding of the 9-exon expansion in *T. fiebrigi* could facilitate CHC recognition.

Studies by Wanner *et al*. (2007), Karpe *et al*. (2016), and Slone *et al*. (2017) suggest that the L subfamily in bees and ants are finely tuned to detect queen pheromones, fatty acids, and CHCs. As previously observed by Brand and Ramírez (2017), this lineage from the G02A subfamily was expanded in obligate eusocial bees, including *T. fiebrigi*, compared with orchid and halictid bees (Table 2). The L subfamily OR numbers observed in *T. fiebrigi* (64) and *M. genalis* (0) would reinforce the hypothesis that the L lineage has a key role in detecting volatiles involved in social behavior (Brand and Ramírez 2017). Interestingly, *T. fiebrigi* showed a large L lineage (64 ORs) only surpassed by the ants *A. cephalotes* (69) and *A. echinatior* (66), within the hymenopterans (Zhou *et al*. 2012; Engsontia *et al*. 2015; Brand and Ramírez 2017; Karpe *et al*. 2017; Legan *et al*. 2021). Determining the genomic location of these ORs as well as codon analysis to reveal signatures of positive selection and gene expression studies in different castes will help to understand the role of these receptors in the sensory biology of *T. fiebrigi*.

Coinciding with Brand and Ramírez (2017), the stingless bees present the largest G09C subfamily, which includes the T and U subfamilies proposed by Zhou *et al*. (2012), among the 7 hymenopteran species studied here (Fig. 1 and Table 2). Moreover, the G09C subfamily is larger in stingless bees than in the solitary bees *Dufourea novaeangliae* and *Habropoda laboriosa*, with 8 and 4 receptors, respectively (Karpe *et al*. 2017). The numbers in the T and U subfamilies for the stingless bees were similar to those reported in ants (Zhou *et al*. 2012). However, no functional information about these subfamilies is available so far.

The GUnC subfamily, which belongs to the H subfamily according to Zhou *et al*. (2012), is expanded in both stingless bees, *M. quadrifasciata* and *T. fiebrigi* (Table 2 and Supplementary Fig. 1). Previous genetic analyses suggest that this subfamily may be involved in detecting floral scents, potentially specializing in sensing terpenoids (Claudianos *et al*. 2014; Karpe *et al*. 2016, 2017). For example, the *A. mellifera* receptor *AmelOr151* shows affinity to linalool, while *AmelOr152* responds to neral, myrcene, and 6-methyl-5-hepten-2-one (Claudianos *et al*. 2014), all compounds commonly found in floral scents (Knudsen *et al*. 2006). Therefore, *T. fiebrigi* ORs from the GUnC subfamily could be linked to the detection of compounds with biological value within the foraging context, such as floral or nectar volatiles (Raguso 2004) related to the presence of flower rewards.

In addition, Karpe and collaborators (2017) proposed that the size of the H subfamily (composed of the GUnC, G02B, and G09A subfamilies) in bees depends on diet profile, being larger in generalists than in specialist pollinators. They observed that 2 specialist bee pollinators, *D. novaeangliae* (3 ORs) and *H. laboriosa* (5 ORs), had a lower number of ORs compared with generalist bees, like *A. mellifera* (13 ORs), *B. terrestris* (11 ORs), or *E. dilemma* (19 ORs). Considering that stingless bees can pollinate many tropical and subtropical plants, including coffee, citrus, and avocado (Heard 1999; Vossler *et al*. 2018; Grüter 2020), our results with *T. fiebrigi* (20 ORs in the H subfamily) and those from *M. quadrifasciata* (22 ORs) point in the same direction as Karpe *et al*. (2017). Interestingly, the stingless bees have the largest H subfamily among hymenopterans (Zhou *et al*. 2012; Engsontia *et al*. 2015; Brand and Ramírez 2017; Karpe *et al*. 2017; Legan *et al*. 2021), transforming this group of receptors into relevant candidates to perform genetic studies in the future.

As was observed in other hymenopterans (Table 1), the GR repertoire of *T. fiebrigi* is much lower than the ORs (9 GRs vs 204 ORs). Robertson and Warner (2006) proposed that the reduced GR repertoire in honeybees was induced by their mutualistic relationship with plants, as these insects have a reduced risk of encountering toxins in their primary food sources (nectar and pollen), which usually drive taste diversity in insects. In addition, bees use their antennae to touch and smell, potentially replacing the need for dedicated taste receptors. Both hypotheses could explain the low number of GRs observed in *T. fiebrigi*. In the honeybee *A. mellifera*, 3 sugar receptor genes were identified: *AmelGr1*, *AmelGr2*, and *AmelGr3* (Robertson and Wanner 2006; Jung *et al*. 2015). *AmelGr1* responds to sucrose, glucose, trehalose, and maltose but not to fructose (Jung *et al*. 2015). The coexpression of *AmelGr1* and *AmelGr2* shows a higher sensitivity to glucose and lower sensitivity to sucrose, trehalose, and maltose compared with *AmelGr1* expression alone (Jung *et al*. 2015), while *AmelGr3* responds to fructose (Değirmenci *et al*. 2023). The orthologs of these GRs were identified in the *T. fiebrigi* database, and they could be involved in sucrose detection that was previously described for this insect (Balbuena and Farina 2020).

The IR coreceptors (*Ir25a*, *Ir8a*, and *Ir76b*) and the conserved antennal IRs, *Ir68a* and *Ir93a*, were identified in *T. fiebrigi*. Studies in *D. melanogaster* have shown that several IRs, including *Ir93a*, *Ir25a*, and *Ir68a*, are needed for sensing changes in environmental humidity and temperature (Enjin *et al*. 2016; Knecht *et al*. 2016, 2017). In this sense, Iwama (1977) found that the foraging activity of the stingless bee *Tetragonisca angustula*, besides light and temperature, is also affected by the relative humidity of the environment. The major activity occurs when humidity is around 30–50%. A similar result was found for *Plebeia pugnax* Moure (in litt.) (Hilário *et al*. 2001). Therefore, there may be a conserved role and some of the mentioned IRs are acting as hygro- or thermoreceptors in *T. fiebrigi*.

In addition, 4 conserved and isolated *Ir75* orthogroups were found for all the bee species analyzed here (Fig. 3). Interestingly, members of the Ir75 clade have been involved in the detection of aliphatic amines and short-chain fatty acids in *D. melanogaster* (Prieto-Godino *et al*. 2017) and *Anopheles gambiae* (Pitts *et al*. 2017). The presence of these receptors in *T. fiebrigi* could be involved in the detection of nitrogen-containing compounds and fatty acid derivates in the flowers (e.g. nectar, floral volatiles) they visit (Muhlemann *et al*. 2014). Regarding the divergent IRs (receptors with numbers higher than 100), any information about their ligands is available so far.

*Megalopta genalis* and *L. albipes* showed similar gene numbers in the GR and IR families (Table 1). In the case of the ORs, *M. genalis* has fewer receptors than *L. albipes* (130 vs 158), and this was due to the contractions observed in the G04A (18 ORs vs 40 ORs), G015A (0 vs 9 ORs), and G02A (1 ORs vs 25 ORs) subfamilies for *M. genalis* (Table 2). Understanding why these particular OR subfamilies were contracted in *M. genalis* requires further studies.

*Tetragonisca fiebrigi* sensory receptors with low expression (e.g. GRs) or those expressed under different physiological conditions may not be present in our database. We could not fully assemble several transcripts, and genomic information will be needed to complete and validate their sequences. Additionally, only once the genomic sequence and gene annotation of *T. fiebrigi* are available can we determine whether transcripts with very similar sequences originate from the same gene (isoforms or allelic variants) or from different genes altogether. The transcriptomic data provided here represent a resource for future molecular and physiological studies in *T. fiebrigi*. The sequences and annotations generated for sensory genes will allow us to perform further experiments to study specific genes and propose functional roles to connect to the sensory physiology of this insect.

## Supplementary Material

jkae060_Supplementary_Data

## Data Availability

The raw sequencing data set is available at SRA with the BioProject number PRJNA1021589: https://www.ncbi.nlm.nih.gov/bioproject/PRJNA1021589. The Transcriptome Shotgun Assembly (TSA) project has been deposited at DDBJ/ENA/GenBank under the accession GKSL00000000. The version described in this paper is the first version, GKSL01000000. Supplemental material available at G3 online.
