# Correction to: Identification of chemosensory genes in the stingless bee *Tetragonisca fiebrigi*

**DOI:** 10.1093/g3journal/jkaf206

**Published:** 2025-09-16

This is a correction to: María Sol Balbuena, Jose M Latorre-Estivalis, Walter M Farina, Identification of chemosensory genes in the stingless bee *Tetragonisca fiebrigi*, *G3 Genes|Genomes|Genetics*, Volume 14, Issue 5, May 2024, jkae060, https://doi.org/10.1093/g3journal/jkae060

The following changes have been made to the originally published manuscript. Mark-up and statements of equal contribution have been added for co-authors on this paper.

