# Supplementary material for: Identification of chemosensory genes in the stingless bee Tetragonisca fiebrigi
Source: G3 (Bethesda). 2024 Mar 18;14(5):jkae060. doi: 10.1093/g3journal/jkae060 (PMC11075565; doi:10.1093/g3journal/jkae060)
Supplement: jkae060_Supplementary_Data [file jkae060_supplementary_data.zip › Supplemental_Material_Legends_G3-2024-404961.docx]

**Supplementary material legends**

**File S1** - Fasta protein sequences of ORs, GRs, and IRs of *T. fiebrigi* and other insects used in the phylogenetic analyses.

**Figure S1 -** **Odorant receptor phylogenetic tree.** The maximum-likelihood tree was constructed using IQ-Tree and bootstrap support corresponds to aLRT-SH values. Subfamilies were identified following Brand and Ramirez (2017) and Zhou et al. (2015) classifications (see Table 2 for further details). The tree was rooted using the Orco clade. Amel, *Apis mellifera*; Bter, *Bombus terrestris*; Edil, *Euglossa dilemma*; Lalb, *Lasioglossum albipes*; Tfie, *Tetragonisca fiebrigi*; Mgen, *Megalopta genalis* and Mqua, *Melipona quadrifasciata*. Sequences with TRINITY IDs belong to *T. fiebrigi.*

**Table S1** - Description of transcripts, annotation, manual curation, the top results of BLASTp searches against SwissProt/UniProt, the output of hmmscan searches by HMMER (Pfam column), number of predicted transmembrane domains by TMHMM, their associated GO and KEGG terms, nucleotide and protein sequences for *T. fiebrigi* ORs (A), GRs (B), and IRs (C).
